# Plasma folate levels are associated with the lipoprotein profile: a retrospective database analysis

**DOI:** 10.1186/1475-2891-9-31

**Published:** 2010-07-28

**Authors:** Alexander Semmler, Susanna Moskau, Andreas Grigull, Susan Farmand, Thomas Klockgether, Yvo Smulders, Henk Blom, Bernd Zur, Birgit Stoffel-Wagner, Michael Linnebank

**Affiliations:** 1 University Zurich, Department of Neurology, Switzerland; 2 University Bonn, Department of Neurology, Bonn, Germany; 3 University Bonn, Department of Clinical Biochemistry and Pharmacology, Germany; 4 VU University Medical Center, Department of Internal Medicine and Metabolic Unit, Amsterdam, The Netherlands

## Abstract

**Background:**

Several studies demonstrated an association of homocysteine plasma levels and the plasma lipoprotein profile. This cross-sectional pilot study aimed at analyzing whether blood levels of the two important cofactors of homocysteine metabolism, folate and vitamin B12, coincide with the lipoprotein profile.

**Methods:**

In a retrospective single center approach, we analyzed the laboratory database (2003-2006) of the University Hospital Bonn, Germany, including 1743 individuals, in whom vitamin B12, folate and at least one lipoprotein parameter had been determined by linear multilogistic regression.

**Results:**

Higher folate serum levels were associated with lower serum levels of low density lipoprotein cholesterol (LDL-C; Beta = -0.164; p < 0.001), higher levels of high density lipoprotein cholesterol (HDL-C; Beta = 0.094; p = 0.021 for trend) and a lower LDL-C-C/HDL-C-ratio (Beta = -0.210; p < 0.001). Using ANOVA, we additionally compared the individuals of the highest with those of the lowest quartile of folate. Individuals of the highest folate quartile had higher levels of HDL-C (1.42 ± 0.44 mmol/l vs. 1.26 ± 0.47 mmol/l; p = 0.005), lower levels of LDL-C (3.21 ± 1.04 mmol/l vs. 3.67 ± 1.10 mmol/l; p = 0.001) and a lower LDL-C/HDL-C- ratio (2.47 ± 1.18 vs. 3.77 ± 5.29; p = 0.002). Vitamin B12 was not associated with the lipoprotein profile.

**Conclusion:**

In our study sample, high folate levels were associated with a favorable lipoprotein profile. A reconfirmation of these results in a different study population with a well defined status of health, diet and medication is warranted.

## Background

Increased homocysteine (Hcys) levels and altered plasma lipid levels are generally considered to be independent risk factors for the development of cardiovascular disease (CVD). However, lifestyle and nutrition may cause a coincidence of the homocysteine and lipoprotein status. Although of potential clinical and scientific relevance such a relationship is not generally acknowledged.

Recent studies have already described an inverse relationship between folate and high-density lipoprotein cholesterol (HDL-C) levels and low folate levels have therefore been suggested to be a cardiovascular risk factor and that the subjects with lower folate levels should be recommended for dietary folic acid supplementation to HDL-C levels [1,2].

Several studies have proposed a direct biochemical link between the lipoprotein and homocysteine metabolism. In rats with experimentally induced hyperhomocysteinemia, plasma cholesterol levels increase, probably because of increased expression and activity of hepatic HMG-CoA reductase, a rate-limiting enzyme in cholesterol biosynthesis [3-5]. Homocysteine supplementation leads to an inhibition of phospholipid methylation and triacylglycerol accumulation in yeast [6], and diet-induced hyperhomocysteinemia leads to cholesterol and triacylglycerol accumulation in mouse liver [7]. The nutritional intake of folate and, to a lower extent, of vitamin B12 are major determinants of plasma homocysteine levels [8]. In wildtype and APOE-deficient mice, depletion of dietary folate leads to increased levels of serum and liver cholesterol and to an altered expression profile of the cholesterol biosynthesis pathway [9].

This study aimed at testing the correlation of plasma levels of vitamin B12 and folate and lipoproteins in a large sample.

## Methods

In a retrospective mono-centered approach, we analyzed the routine laboratory database (2003-2006) of the University Hospital Bonn, Germany using data that were made anonymous. We selected all in- and outpatients in whom vitamin B12 and folate as well as lipoproteins had been determined (table). Serum triglycerides (TG), total cholesterol (TC), high density lipoprotein cholesterol (HDL) and low density lipoprotein cholesterol (LDL-C) were measured on the Dimension RxL clinical chemistry system (Dade Behring, Schwalbach, Germany) with enzymatic methods using the manufacturer's reagents and instructions.

Hcys was determined by means of fully automated particle-enhanced immunonephelometry with a BN II System (Dade Behring). Bound homocysteine in the heparinized plasma sample is reduced to free homocysteine by the action of dithiothreitol, and then converted enzmatically to S-adenosyl-homocysteine (SAH) in the next step. Conjugated S-adenosyl-cysteine (SAC), added at the onset of the reaction, competes with SAH in the sample for bonding by anti-SAH antibodies bound to polystyrene particles. The result is evaluated by comparison with a standard of known concentration.

Serum concentrations of vitamin B12 and folate were measured by means of a competitive chemiluminescent immunoassay with an Accessâ„¢ Immunoassy System (Beckman Coulter, Krefeld, Germany) according to the manufacturer's instructions.

Due to anonymous data extraction, information on diagnosis and diet were not available.

Because some parameters were not normally distributed, log transformation was performed for statistical analysis. Multiple linear regression analysis with α = 0.01 (multiple testing of four different lipoproteins) was used to simultaneously analyze independent associations of age, gender, folate and vitamin B12 on the lipoprotein levels as dependent variables. We used stepping mode criteria with a probability of 0.05 for entry and of 0.10 for removal from the equation. Due to the low number of patients for whom homocysteine was known, homocysteine was not included as covariable in the multivariate model. Instead, bivariate Pearson's analysis was calculated. The lipoproteins values of folate and vitamin B12 quartiles were compared by ANOVA. Since this analysis was done following the results of the regression analysis, we did not correct for multiple testing (α = 0.05).

## Results

1743 individuals were included of whom age (mean age ± standard deviation: 46 ± 17), gender (45% female), plasma folate levels and plasma vitamin B12 levels were known. The number of available values differed between the lipoprotein parameters (table 1). As shown in table 2, higher folate levels were associated with lower levels of LDL-C, a lower LDL-C/HDL-C-ratio, and, for trend, higher HDL-C levels. Folate was associated with vitamin B12 (Beta = 0.223; p < 0.001). However, vitamin B12 was not associated with the lipoprotein profile. Using ANOVA, we additionally compared the individuals of the highest with those of the lowest quartile of folate and vitamin B12 levels and found that individuals in the highest folate quartile had a 1.53-fold lower LDL-C/HDL-C-ratio in comparison to those in the lowest folate quartile (table 3), whereas quartiles of vitamin B12 levels showed no significant association with lipoprotein levels.

**Table 1:** Laboratory parameters included in this study

parameter	individuals analyzed	mean value	standard deviation	median value	range	reference range
folate (nmol/l)	1743	14.94	8.53	12.21	2.18-44.61	6.8-39

vitamin B12 (pmol/l)	1743	215	147	255	60-1053	130-675

Homocysteine (Âµmol/l)	123	13.6	4.4	12.5	6.8-30.6	<16

LDL-C (mmol/l)	593	3.34	1.05	3.28	0.21-7.81	<4.10

HDL-C (mmol/l)	593	1.40	0.49	1.34	0.10-3.36	1.0-1.55

total cholesterol (mmol/l)	123	5.61	1.08	5.59	2.74-8.92	<6.20

triglycerides (mmol/l)	1468	1.56	1.49	1.26	0.25-30.46	<1.70

**Table 2:** Effect of age, gender, folate and vitamin B12 on lipoprotein levels

	triglycerides	HDL-C	LDL-C	LDL-C/HDL-C	total cholesterol
n	1468	593	593	592	123

Folate	Beta = -0.045; p = 0.087	Beta = 0.094; p = 0.021	Beta = -0.164; p < 0.001	Beta = -0.210; p < 0.001	Beta = -0.138; p = 0.147

Vitamin B12	Beta = -0.051; p = 0.052	Beta = 0.030 p = 0.458	Beta = 0.070; p = 0.094	Beta = 0.058; p = 0.164	Beta = -0.151; p = 0.110

Age (year)	Beta = 0.125; p < 0.001	Beta = -0.012; p = 0.762	Beta = 0.254; p < 0.001	Beta = 0.160; p < 0.001	Beta = 0.178; p = 0.054

Gender (male)	Beta = 0.211; p < 0.001	Beta = -0.352; p < 0.001	Beta = -0.082; p = 0.045	Beta = 0.186; p < 0.001	Beta = -0.180; p = 0.052

Linear logistic regression analysis (standardized partial regression coefficient Beta and p-value) of the independent predictive character of the covariables age, gender, plasma folate level, and plasma vitamin B12 level for the dependent variables triglycerides, HDL-C, LDL-C, LDL-C/HDL-C-ratio and total cholesterol levels are depicted. As these were five separate analyses, we set the level of significance to alpha = 0.01

**Table 3:** Laboratory parameters of individuals of the highest and lowest quartiles of folate levels.

	high folate (≥18.75 nmol/l)	low folate (≤8.75 nmol/l)	ANOVA
vitamin B12 (pmol/l)	334 ± 154 (n = 407)	260 ± 145 (n = 416)	p < 0.001

Triglycerides (mmol/l)	1.47 ± 0.94 (n = 334)	1.66 ± 1.82 (n = 388)	p = 0.075

HDL-C (mmol/l)	1.42 ± 0.44 (n = 170)	1.26 ± 0.47 (n = 87)	p = 0.005

LDL-C (mmol/l)	3.21 ± 1.04 (n = 167)	3.67 ± 1.10 (n = 87)	p = 0.007

LDL-C/HDL-C-ratio	2.47 ± 1.18 (n = 167)	3.77 ± 5.29 (n = 87)	p < 0.001

Total cholesterol (mmol/l)	5.43 ± 0.97 (n = 36)	5.77 ± 1.18 (n = 19)	p = 0.346

Linear logistic regression analysis (table 2) revealed that male gender was associated with higher levels of triglycerides, lower levels of HDL-C, and a higher LDL-C/HDL-C ratio. In bivariate Pearson's analysis, homocysteine correlated with folate (Pearson = -0.408, p < 0.001) and vitamin B12 (Pearson = -0.0228, p = 0.011), but not with lipoprotein parameters. However, respective data were only available for n = 63 (homocysteine and triglycerides) and n = 123 (homocysteine and cholesterol; details not shown) patients. Higher age was associated with higher levels triglycerides, higher levels of LDL-C, a higher LDL-C/HDL-C ratio (table 2).

## Discussion

In a retrospective database analysis we confirmed that male gender and older age were associated with more unfavorable lipoprotein profiles. More important, we also observed that higher levels of folate were associated with a favorable lipoprotein profile independently of the covariables age and gender. Vitamin B12 levels did not influence the lipoprotein profile in our study sample. As limitation, our study made use of an unselected mono-centered anonymous patient sample that cannot be representative for any general population. The available set of lipoprotein parameters differed between the enrolled samples and the results cannot be controlled for any influence of drugs, diseases, physical exercise, and diet or body mass index. Thus, the significance and the effect size of folate levels for the lipoprotein profile should additionally be analyzed in samples of individuals of defined clinical and dietary status as our data raise the question whether high folate levels and favorable lipoprotein profiles coincide due to nutritional reasons or a "healthy lifestyle". However, the association between the folate and the plasma lipoprotein profile observed in this pilot study suggests that such an interaction may be a relevant confounder for effects of folate or lipids, respectively, on vascular disease. Thus, both parameters should be analyzed in multivariate approaches in respective studies.

The differences in the levels of LDL-C, HDL-C and LDL-C/HDL-C ratio observed in subjects with high or low levels of folate should be physiologically relevant. In a large prospective study including 12339 participants it was shown that a one standard deviation (1 mmol/L) increase in LDL above the mean of 3.50 mmol/L is associated with an age adjusted relative risk for coronary heart disease of 1.42 for men and 1.37 for women. A one standard deviation increase in HDL (0.40 mmol/L) above the mean of 1.18 mmol/L in men and 1.51 mmol/L in women was associated with an adjusted relative risk of 0.64 and 0.69, respectively [10]. Although the differences in HDL and LDL levels in high and low folate quartiles where not as high as one standard deviation, our data demonstrate that folate levels have a relevant influence on HDL and LDL levels in addition to other factors such as age and gender.

Additionally it is tempting to speculate about a possible direct biochemical link between the plasma lipoprotein and the folate metabolism. Our dataset did not allow powerful analyses of the association of homocysteine with lipoprotein parameters. Therefore, it is not possible to decide, whether the homocysteine lowering effect of folate or a direct effect of folate is statistically more likely to explain any association between folate and lipid levels. Folate is transported into cells by two distinct transport mechanisms. The first is a low affinity system called reduced folate carrier 1 (RFC1). The second system is a high affinity system that involves phagocytosis of folate by membrane bound folate receptors. When folate levels are low, the membrane bound folate receptors are used [11]. Previous studies showed that cholesterol regulates the rate of cellular folate import by facilitating the clustering of membrane bound folate receptors on the cell membrane [12-14]. Fibroblasts from patients that are unable to produce sufficient cholesterol levels show decreased folate import [15]. Thus, the observed association of folate plasma levels and the lipoprotein profile may be based on both nutritional and biochemical factors. Studies investigating whether medical manipulation of the lipoprotein profile impacts folate metabolism and whether folate substitution is relevant for the lipoprotein profile may be interesting.

## Conclusion

A higher level of folate is associated with a favorable lipoprotein profile independently of the important covariables age and gender. A reconfirmation of these results in different study populations with a well defined status of health, diet and medication may help to clarify whether the lipoprotein and folate status coincide due to nutritional reasons or due to a biochemical link between folate and lipoprotein metabolism.

## Competing interests

The authors declare that they have no competing interests.

## Authors' contributions

AS, SM, and ML designed the study, performed statistical analysis and wrote the manuscript.

AG, SF, TK, BZ, and BSW collected the data, helped with data analysis and contributed to finalisation of the manuscript. YS, and HB contributed to the study design and manuscript preparation.

All authors read and approved the final manuscript.
